# Numerical Simulations Reveal Randomness of Cu(II) Induced Aβ Peptide Dimerization under Conditions Present in Glutamatergic Synapses

**DOI:** 10.1371/journal.pone.0170749

**Published:** 2017-01-26

**Authors:** Wojciech Goch, Wojciech Bal

**Affiliations:** Institute of Biochemistry and Biophysics, Polish Academy of Sciences, Warsaw, Poland; University of Akron, UNITED STATES

## Abstract

The interactions between the Aβ_1–40_ molecules species and the copper ions (Cu(II)) were intensively investigated due to their potential role in the development of the Alzheimer Disease (AD). The rate and the mechanism of the Cu(II)—Aβ complexes formation determines the aggregation pathway of the Aβ species, starting from smaller but more cytotoxic oligomers and ending up in large Aβ plaques, being the main hallmark of the AD. In our study we exploit the existing knowledge on the Cu(II)—Aβ interactions and create the theoretical model of the initial phase of the copper- driven Aβ aggregation mechanism. The model is based on the direct solution of the Chemical Master Equations, which capture the inherent stochastics of the considered system. In our work we argue that due to a strong Cu(II) affinity to Aβ and temporal accessibility of the Cu(II) ions during normal synaptic activity the aggregation driven by Cu(II) dominates the pure Aβ aggregation. We also demonstrate the dependence of the formation of different Cu(II)—Aβ complexes on the concentrations of reagents and the synaptic activity. Our findings correspond to recent experimental results and give a sound hypothesis on the AD development mechanisms.

## Introduction

The association between the overproduction and subsequent aggregation of Aβ peptides (Aβ_1–40_ and Aβ_1–42_) in the brain and the Alzheimer’s Disease (AD) has been documented convincingly for both the familial (inherited) and the dominant sporadic type of the disease [1]. The extracellular deposits of aggregated Aβ (senile plaques) are the most profound hallmark of AD. The plaques, which also contain surprisingly high amounts of Cu(II) and Zn(II) ions [2], were initially considered to convey neurotoxicity of Aβ peptides, but later results indicated small Aβ oligomers to be key toxic species [3–6], with plaques likely serving a neuroprotective purpose as relatively safe Aβ dumps. The toxicity of oligomers comprises inhibition of the long term potentiation (LTP) of synapses, increase of neuronal membrane permeability, deactivation of glutamate receptors and eventually neuronal death [7–12].

The formation of Aβ aggregates was investigated by numerous authors. The peptides generally remain monomeric at submicromolar concentrations at neutral pH; the limiting concentrations above which precipitation occurs are 14 and 2 μM for the most frequently investigated Aβ_1–40_ and Aβ_1–42_ peptides, respectively [13–15]. Forms assumed by these peptides in the course of aggregation include oligomers, fibrils and eventually amyloid plaques [16–18]. Interactions with Cu(II) and Zn(II) ions also result in the formation of amorphous aggregates [19,20]. Among these species, the dimers are logically assumed to be the simplest aggregated Aβ forms, and thus the common initiating species for all aggregation pathways [16,17]. The Aβ dimers were indeed shown to be highly toxic [6,21] and stable at nanomolar concentrations [22].

Aβ_1-x_ peptides, including Aβ_1–40_ and Aβ_1–42_ bind Cu(II) predominantly with an equimolar stoichiometry. The resulting complexes have the conditional stability constant of the order of 10^10^ M^-1^ at pH 7.4 [23,24]. The exact value for Aβ_1–40_ is 2.7 × 10^10^ M^-1^ [23] and the same value is assigned to the Aβ_1–42_ peptide. The pleiomorphic Cu(II) binding site is localized in the N-terminal part of the peptide, with the involvement of the first two N-terminal residues (Asp1, Ala2) and imidazoles of His6, His13, and/or His14 side chains [25,26]. Kinetic studies suggest that the rate of formation of this Cu(II)-Aβ complex is close to the diffusion limit, i.e. its value can be estimated to be in the range of 10^9^–10^10^ Ms^-1^ [27,28].

Cu(II) ions accelerate the Aβ aggregation *in vitro* by three to four orders of magnitude—from hours/minutes, depending on the individual Aβ_1-x_ peptide, to seconds [29,30]. The stoichiometry of Cu(II)—Aβ interactions relevant to the aggregation phenomena is under intense debate. As mentioned above, the monomeric Aβ peptides form predominantly 1:1 complexes, with some evidence of Cu(Aβ)_2_ [27,31] and Cu_2_(Aβ) forms [26,32]. The 1:1 stoichiometry is also predominant in fibrils [5], although recent findings indicate that interactions between neighboring Aβ chains result in a significant increase of Cu(II) affinity in this case [17,33].

**Fig 1:**
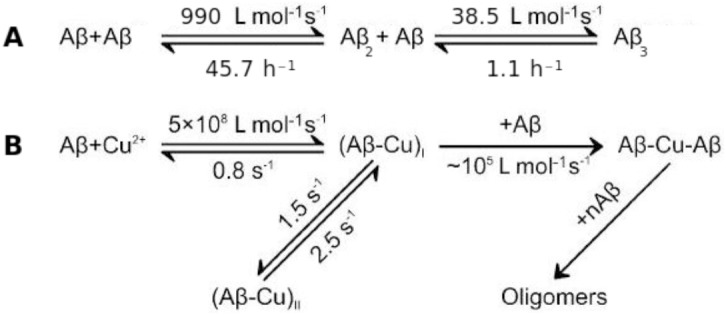
The models of interactions adopted in this study. (A) Adopted after Garai and Frieden ([34]). (B) Adopted after Branch et al. [27].

On the basis of fluorescence, stopped-flow and NMR relaxation studies Pedersen et al. suggested that the CuAβ_2_ complex is present transiently in solution at a very low concentration [31]. Very recently, Branch et al. [27] performed a thorough stopped-flow kinetic study of Cu(II) binding to Aβ_1–16_ and Aβ_1–40_ peptides at pH 7.4, proposing the mechanism of interactions leading to the formation of the CuAβ_2_ complex, considered as a gateway to (Cu)_x_Aβ_n_ oligomer formation. They also established kinetic parameters for all complex formation steps in their model of interactions (Fig 1).

Concluding from fluorescence quenching experiments, Branch et al. proposed the existence of two conformers of the CuAβ complex, where conformer I is formed initially and has a potential second Aβ binding site, whereas conformer II lies on an off-pathway (from hereafter denoted as CuAβ I and CuAβ II, respectively). The presence of two CuAβ conformers in physiological conditions was already reported in a number of structural studies [26,35,36]. The model of Branch et al. was adopted in our analysis presented below.

The initial Cu(II)/Aβ ratio is a crucial factor determining the pathway of aggregation and its final form [20,31,37]. Various scenarios were postulated for different conditions, including the formation of covalent cross–links between monomers in equimolar conditions [38], but in general, the Cu(II) presence leads to amorphous aggregates [19], perhaps due to a disruption of intermolecular β-sheets [39].

**Fig 2:**
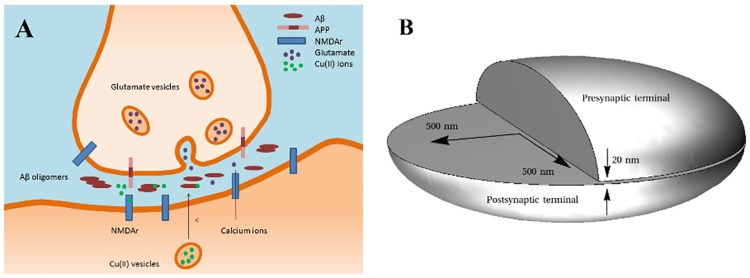
Schematic illustration of the role of Cu(II) ions in Aβ aggregation mechanism (A) and visualization of actual proportions of dimensions of the synaptic cleft (B).

The oligomerization of Aβ peptides takes place in synapses, structures composed by endings of two different neurons, physically separated but functionally connected by the synaptic cleft [9,40,41]. The synaptic cleft is the venue for exchange of chemical signals between the neurons which constitute neurotransmission and neuromodulation. The synaptic cleft is flanked by non-neuronal brain cells (microglia, astrocytes), and thus can be considered as a confined biological compartment. Two of its dimensions, provided by cell membranes of the neurons (width and breadth) are relatively large (~μm), while the third, constituting the distance between the neurons (depth) is very small (~20 nm) (Fig 2B). The volumes of synaptic clefts in the human brain generally range from 2 to 20 aL [42]. Indeed, the synaptic cleft is one of the places where Aβ is initially released upon the β-secretase cleavage of its precursor protein APP (in addition to ER, Golgi apparatus and endocytic pathway [43–45]), and consequently it is the presumable site of Aβ peptide oligomerization and metal—Aβ peptide interactions [17,46,47].

In systems where the volume of the chemical reaction compartment is small and/or substrate concentrations are very low, the probabilistic nature of chemical reactions, resulting from the stochastic occurrence of intermolecular collisions, may play a significant role. When the average concentration of a chemical species corresponds to only a few molecules, the intrinsic noise (unpredictability of evolution of the system of interacting molecules) becomes a substantial or sometimes even dominant factor in the system. This question was already addressed in several papers [48–50], but not in the context of chemical reactions taking place in extremely small volumes, such as synaptic clefts. The acknowledged method of deriving theoretical prediction for noise–driven systems adopted in this work is to formulate the adequate mathematical model, given in terms of probabilistic functions and described by the well-established Chemical Master Equation (CME) [51]. The Gillespie Algorithm, popularized by Dan Gillespie in the late 1970-ies, is an algorithm for acquiring exact sample trajectories for the evolution of such systems.

Our choice of CME model was dictated by the fact that such approach constitutes a formal way to incorporate a randomness arising from the aforementioned discreteness of our system. Such approach is distinct from other simulations, which adopt Molecular Dynamics (MD) to investigate the conformation of Aβ aggregates (reviewed in [52]) or use theoretical models described in terms of differential equations (DE) to study the kinetics of the aggregation process [53,54]. Our model shares some similarities with the later approach. First of all, to our best knowledge it is the first model to quantitatively estimate complex formation upon interactions of Aβ peptides with Cu(II) ions. Secondly, instead of tracking the change in the concentration (which is characteristic for DE) our model calculates the distribution of probabilities for all possible outcomes of the reactions at every time point. The strength of such modeling technique lies in fact that, apart from the general kinetics of the model, we estimate the intrinsic variability (stochastic noise) of the system and incorporate the dependence of the system evolution on the volume of the synaptic cleft. Such approach is limited by a lack of structural information (on the single–molecule level) and by not taking into account the dependence on morphological shape of the cleft (on the level of the whole system).

**Fig 3:**
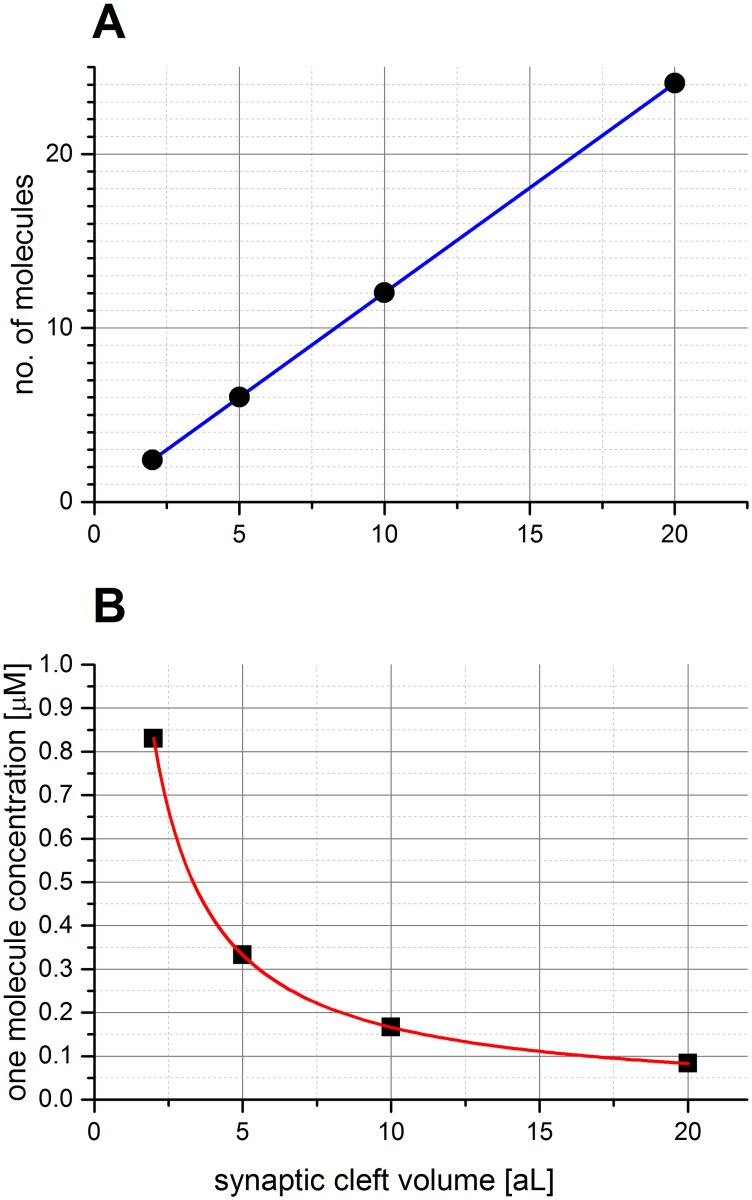
The correlation between the number of molecules, concentrations and the range of synaptic clefts. (A) The number of Aβ_1–42_ molecules corresponding to the aggregation concentration (2 μM) in the volume range of synaptic clefts. (B) The correlation between the synaptic cleft volume and the formal concentration corresponding to the presence of just one molecule of a given species.

Straightforward calculations lead to the apparently overlooked, but crucial conclusion that the attoliter volume of synaptic clefts is so small that micromolar or nanomolar concentrations correspond to very low or even fractional (thus non-physical) numbers of molecules, as illustrated in Fig 3.

The subnanomolar concentrations reported in the literature for Aβ peptides in the brain are based on the cerebrospinal fluid analyses (CSF) [13,14] and on the interstitial fluid microdialysis [15]. As illustrated in Fig 2B, such concentrations are irrelevantly low for synaptic clefts. Consequently, it only takes a dozen or less molecules to reach the concentration sufficient for spontaneous Aβ aggregation as observed in test tube experiments (Fig 3A).

A vast number of studies showed that the accumulation of Aβ peptides in AD affected patients is accompanied with the decreased levels of Aβ_1–42_ in CSF [55,56] which in turn is associated with the formation of amyloid plaques [57]. This is consistent with findings that the level of Aβ oligomers is elevated in AD [58]. Moreover, the cleavage of APP is amplified due to increase in the activity of the β–secretase protein [59], with not a fully defined role of presenilin [60,61]. On the level of a single synapse the following picture emerges—in a healthy brain the numbers of Aβ molecules oscillate around one molecule, which already constitutes a reported physiological concentration. In AD the presented observations suggest that this numbers are probably higher, firstly because the increased Aβ production directly elevates the numbers of molecules in the synapse and secondly because the significant presence of soluble oligomers automatically requires an adequate amount of Aβ monomers.

As stated above, the Aβ aggregation is accelerated by the presence of Cu(II) ions. The release of Cu(II) ions from the postsynaptic terminal is correlated with the neurotransmitter release after excitation of the presynaptic ending. Cu(II) ions can reach a peak concentration in the synaptic cleft as high as 100–250 μM [62–64]. Then they are cleared rapidly (on a millisecond timescale) to help the synapse recover for the next impulse. The Cu(II) uptake is mainly due to the hCtr1 membrane copper receptor [65] present inside the synaptic cleft (on neurons) and outside the cleft (on glial cells) and to metallothionein-3 in the extracellular space [66]. The reported levels of Cu(II) ions in CSF are on the level of 0.2–0.5μM [67,68], which corresponds to only 1–6 ions inside the synaptic cleft, depending on the volume(Fig 3), whereas the very high, but temporal elevation of the Cu(II) concentration results from the release of approximately 300 ions in a typical (5aL) synapse. The additional observation is that the presence of one ion constitutes a minimal (nonzero) concentration, i.e. 83 nM for largest synapses (20 aL) and 830 nM for the smallest ones (2 aL).

In this work we investigated the possible course of the very first step of Aβ aggregation, i.e. the Aβ dimer formation, and the potential role of Cu(II) ions in this process. We simulated different scenarios of Cu(II) release into the synaptic cleft, tracked the formation of dimers and checked how the behavior of the system depends on concentrations of reagents and volumes in which the modeled process takes place. In order to account for effects arising from a small number of interacting molecules we applied the stochastic description which helped us reveal the importance of the probabilistic factor in the modeled process. In our analysis some arbitrary choices regarding concentrations and timescales had to be made. In particular, we performed calculations for the range of 1 to 10 Aβ molecules, which correspond to high nanomolar to low micromolar concentrations, depending on the synaptic cleft size (see Fig 3). This range covers the predicted number of molecules in both healthy and pathological conditions. The number of Cu(II) ions varied depending on the considered scenario. Also two distinct time scales were analyzed—4 ms and 20 s. The shorter timescale of 4 ms corresponds to the average time of signal transmission and therefore determines the interval between consecutive Cu(II) releases. The longer timescale was chosen in such a way that the Aβ dimerization induced by Cu(II) ions could be covered, according to the set of reaction rates presented in the model of Branch et al. implemented in our work [27].

## Methods

**Fig 4:**
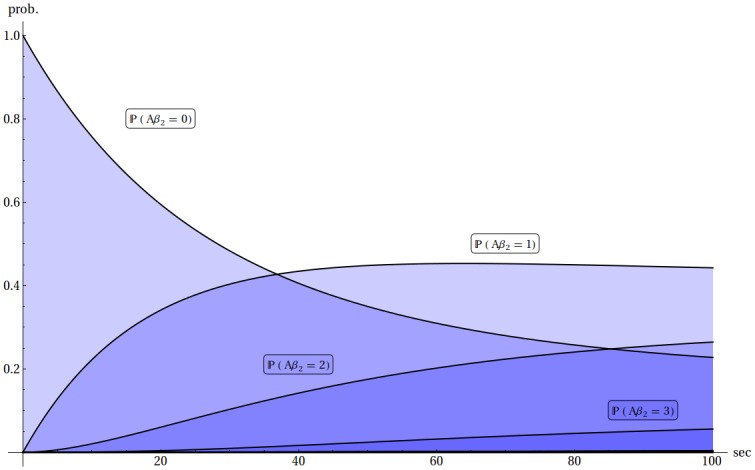
The example of evolution of probability functions. The presented probability functions describe the model of oligomerization of the Aβ_1–42_ peptide when 10 Aβ_1–42_ molecules are present initially in the 5 aL volume.

In our research we applied a space–independent stochastic model described in terms of states and their probabilities. Every state was treated as a set of numbers of molecules of all species in the model. To every state we assigned a probability that the model is in this particular state at a given time t. The evolution of these time dependent probabilities was described by a set of ordinary differential equations derived from the law of mass conservation (CME). A brief account of assumptions and details of the CME approach is given in Supplementary Information, Section 1 (S1 Text). An example of solutions of CME is given in Fig 4. The equations were solved numerically using built-in solvers of the Wolfram Mathematica 9 environment.

**Fig 5:**
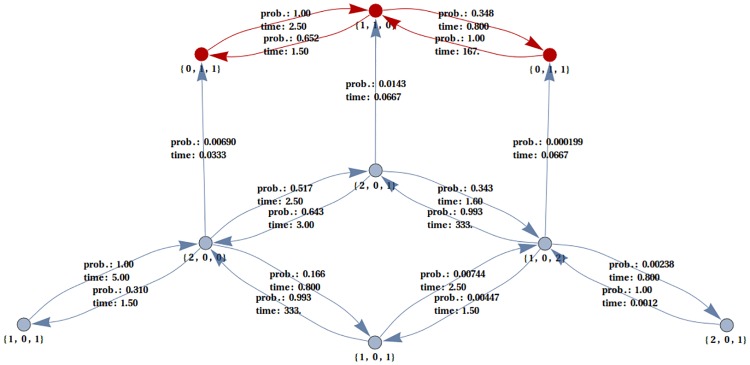
Graph of probabilities and times of passage between states. Every state is defined by numbers of three kinds of molecules: Aβ monomers, CuAβ I complexes and Cu(Aβ)_2_ complexes (here described as indexes of the graph nodes). The system when initially there are 3 Aβ molecules and 2 Cu(II) ions is presented.

Average times until the appearance of a given complex were calculated from a set of algebraic equations relating the times of passage between states to the occurrence of a particular group of states, defined by the first appearance of a molecule of the complex of interest. Such set of equations can be visualized by a corresponding graph, in which vertexes are possible states and edges are weighted by average times of passage between states. An example of such graph with the aforementioned states highlighted in red is presented in Fig 5.

The calculations were first performed for the dimerization of the Aβ_1–42_ peptide alone, and followed by calculations on the Cu(II)-dependent dimerization according to the experimental model by Branch et al. [27]. Three synaptic states: resting state, excited state and strongly stimulated state and four possible scenarios based on these states were considered. In all calculations the number of Aβ molecules ranged from 1 to 10. In the resting state the number of Cu(II) ions also ranged from 1 to 10 and no additional fluxes were included. In the excited state we modeled a single event of Cu(II) ions release, which resulted in an immediate elevation of Cu(II) concentration, followed by a time dependent decrease due to the diffusion of Cu(II) ions outside the cleft (S2 Text). In the third model two scenarios were considered: (a) a repetitive activation followed by a number of releases of Cu(II) ions in regular intervals and (b) a strong excitation immediately followed by a series of succeeding Cu(II) releases. A summary of these computational models of synaptic states is shown in Table 1.

**Table 1:** Summary of synaptic models used in calculations.

Model	Range of Aβ peptide molecule numbers	Range of Cu(II) ion numbers	No of Cu(II) release events	Frequency of Cu(II) release events [1/s]
**1.Resting**	1–10	1–10	N/A	N/A
**2.Excited**	1–10	50–500	1	N/A
**3.(a) Chronic stimulation**	1–10	50–500	10	1/2
**3.(b) Strong stimulation**	1–10	50–500	10	1/(4 × 10^−3^)

We performed our analysis for four physiologically realistic volumes– 2, 5, 10 and 20 attolitres, covering most of this size variability [42].

As stated above, the numbers of molecules interacting in systems studied here are not sufficient for the usage of average parameters, such as concentration, to describe their behavior. Instead, the stochastic calculations were performed. In their course, we determined the distribution of possible outcomes of reactions and their probabilities, depicting an inherent noisiness of the systems. In order to give a uniform characterization of noise, we define relative standard deviation (RSD) as its measure:

$$RSD=\frac{Standard\;deviation}{Expected\;value}$$

RSD shows a relation of variability in a given parameter to its expected (average) value. This measure can be determined for each reacting species separately at every time point. In our work we will discuss only the RSD values for the Aβ dimers (both with and without Cu(II)), because they are in the center of our attention as an entry to the aggregation pathway and likely toxic species on their own.

RSD can be interpreted as a measure of uncertainty defined as a fraction of the expected value. While it is a reasonable measure of noise, it always has to be analyzed in respect to the expected values, since the systems where expected values are close to zero are characterized with the increasing RSD values (S3 Text).

The parameters yielded by our calculations covered the evolution of the average number of molecules, its RSD and the average time until the first appearance of both CuAβ conformers and the CuAβ_2_ complex detected by Branch et al [27].

The average time until the appearance of a given complex were chosen to monitor the system evolution, because of the irreversible nature of Aβ dimerization and because the eventual toxic processes are evoked by these dimers. The latter occurs beyond the kinetic system studied. Within the framework of this study, we can only investigate the ability of the system to generate toxin, on the assumption that its further molecular interactions do not form a feedback loop with the system.

## Results

### Aβ dimerization in the absence of Cu(II)

In the first stage of our calculations a simple model of Aβ_1–42_ aggregation in the absence of Cu(II) ions was taken into account. The model of dimer and trimer formation was adopted from work of Garai and Frieden [34], who determined the rate constants of formation and dissociation for these species. The value of dimerization rate constant from their work (990 M^-1^s^-1^) is consistent with determinations made in other studies [53,54] as well as estimations based on the data for the n-mer formation [69]. The rate of oligomerization is generally higher than the mean rate of aggregation. This probably reflects the change of association mechanism upon the formation of larger aggregates [70].

**Fig 6:**
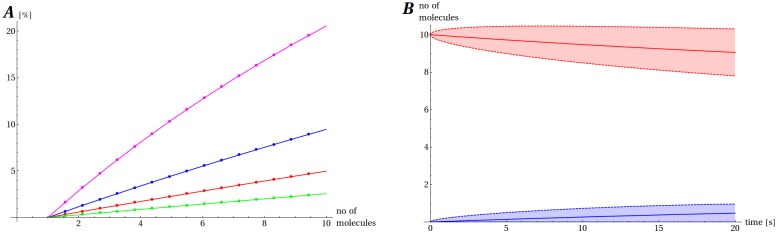
Dimerization of Aβ in the absence of Cu(II). (A) The expected fraction of Aβ_2_ dimer after 5 min in a 2 aL (magenta), 5 aL (blue), 10 aL (red) and 20 aL (green). (B) The expected number of Aβ monomers (red) and Aβ_2_ dimers (blue) together with their standard deviations for a case when 10 Aβ molecules are present initially in a 5 aL volume.

The time until dimerization calculated by us is of the order of minutes and can vary to a large extent depending on conditions. Also the average number of dimers depends on both the initial number of Aβ molecules and the volume (Fig 6A). The fraction of the dimerized peptide increases steadily in the course of this irreversible process, proportionally to the initial number of molecules, and after 20 s situates itself in a wide range between 0.3% (for 20 aL) and 21% (for 2 aL) (S1 Table). The interval of 20 s was too short to observe a substantial number of trimers (fraction of trimers < 0.05% in all cases) and they were excluded from further analysis. The RSD is in the wide range from 0.8 (2 aL, 10 Aβ molecules) to 18.52 (20 aL, 2 Aβ molecules) and for the range of initial conditions that we took into account, its median is 2.97. The relatively high RSD values result from the fact that the average numbers of Aβ_2_ dimers are less than one, which boosts the values for RSD (S3 Text). In this model the formation of dimers is a slow process progressing steadily, but with a significant uncertainty of outcome for a given numerical experiment (noise) involved (Fig 6B).

### The Cu(II)-induced Aβ dimerization in the resting state of the synapse

For this state two cases are considered: (i) when the number of Cu(II) ions is equal to or larger than that of Aβ molecules (Cu(II) ≥ Aβ) and (ii) when it is lower (Cu(II) < Aβ).

#### Cu(II) ≥ Aβ

**Fig 7:**
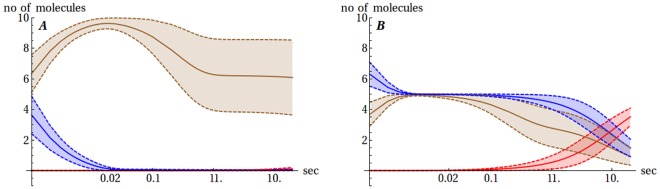
Sample model evolution, cases with different Cu(II) to Aβ ratio. The expected number of Aβ monomers (blue), CuAβ I (brown) and CuAβ_2_ (red) according to the model of Branch et al. [27] together with their standard deviation for a case when 10 Aβ and 10 Cu(II) ions (A) or 10 Aβ and 5 Cu(II) ions (B) are present initially in the 5 aL volume. The time scale is logarithmic. CuAβ II complexes are omitted for the sake of clarity of presentation.

The presence of Cu(II) ions changes the ability of the Aβ peptide to dimerize drastically, but this effect depends on the Cu(II)/Aβ ratio. In cases when Cu(II) ≥ Aβ, the CuAβ I and CuAβ II complexes become the major components of the analyzed system (Fig 7A). Due to a high complex stability, its formation is limited practically only by the Cu(II) availability. The complex formation is very fast—after only four milliseconds 70–90% of total Aβ becomes bound in the CuAβ I complex, depending on the ratio of reagents (S2 Table). Such a short time results from the proximity of the ligands (i.e. small volume of synaptic cleft) and a very high association constant, close to the diffusion limit [27,28]. The chance of CuAβ_2_ appearance is close to zero (~ 5% in most favorable cases, ~ 1% in general) in the short timescale of 4 ms, which is the time of a single synaptic excitation. Similar values are taken in a longer timescale of 20 s (S3 Table), which corresponds to the expected time of CuAβ_2_ appearance under more favorable conditions (see below).

The RSD values for CuAβ_2_ complex ranged from 3 to 57, but the highest RSD values corresponded to the lowest expected values (S3 Text) and consequently the CuAβ_2_ presence in the system was negligible.

#### Cu(II) < Aβ

A different picture appears when Aβ molecules are in excess over Cu(II). In such conditions, the CuAβ I formation is limited by the number of Cu(II) ions while the presence of free Aβ monomers allows for the eventual formation of CuAβ_2_ complexes. The time scale of formation of CuAβ and CuAβ_2_ complexes differs by three orders of magnitude. In the millisecond timescale the system behavior is similar to the former case—all Cu(II) ions bind to Aβ in a 1:1 stoichiometry forming the CuAβ I complex (Fig 7B). The formation of CuAβ II takes more time, in the range of 0.1 to 1 s. Its appearance is connected with the widening of the standard deviation range for CuAβ I (Fig 7). It also lowers the probability of the dimer formation, due to the fact that it is a dead end in the aggregation pathway presented in Scheme 1.

**Fig 8:**
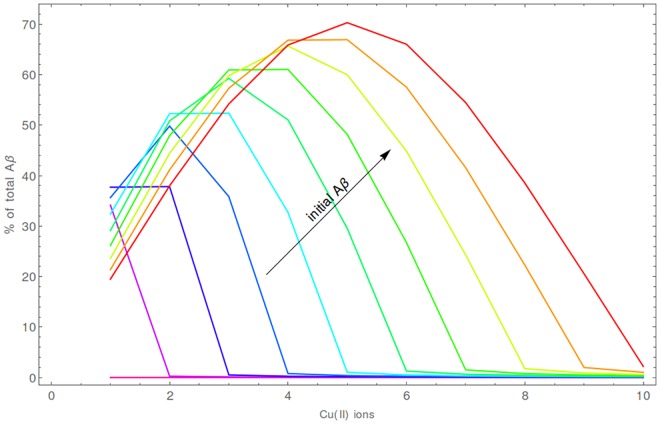
The average fraction of total Aβ bound as a CuAβ_2_ complex [%] for a 5 aL synaptic cleft after 20 s. Results are presented for initial numbers of Aβ molecules ranging from 2 (violet) to 10 (red). The results for CuAβ_2_ in other volumes in are presented in S3 Table and results for CuAβ I after 4 ms in S5 Table.

**Fig 9:**
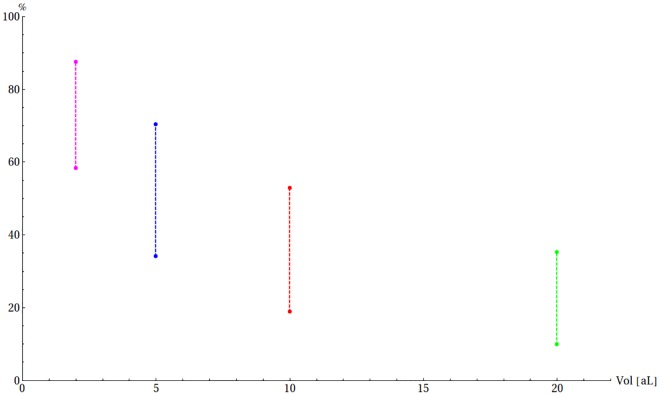
The range of expected amounts of CuAβ_2_ expressed as a fraction of total Aβ after 20 s. The volume of synaptic cleft was 2 aL (magenta), 5 aL (blue), 10 aL (red) and 20 aL (green).

The average time until the formation of CuAβ_2_ is relatively long—seconds, but it still much faster than the formation of Aβ_2_ dimers. The yield of this process, measured by the Aβ percentage, is the highest when the Cu:Aβ ratio is exactly 1:2, reaching 30%—70% in a typical (5 aL) synaptic cleft (Fig 8). A strong volume dependence can be observed for this process. In a 2 aL cleft the total Aβ in the CuAβ_2_ dimer ranges from 58% to 87%, whereas in a 20 aL cleft it does not exceed 35%. (Fig 9, S6 Table).

The RSD for the CuAβ_2_ complex was calculated in a longer timescale of 20 s where the probability of its formation is sufficient for reliable estimations. In such cases, the RSD ranged from 16% to 139% for a typical 5 aL synaptic cleft (S3 Table). The highest values occurred for cases where there were only a few molecules in the system, e.g. two Aβ peptides and one Cu(II) ion (139%) or three Aβ peptides and one or two Cu(II) ions (87% in both). In general, the level of noise was moderate (median of 28%) and higher in cases closer to the 1:2 stoichiometry (median of 49% for cases where the stoichiometry was exactly 1:2). On the other hand, a strong volume dependence could be noticed. Because the extent of dimer formation was higher in smaller synapses, with a simultaneous decrease of the noise, the RSD values were higher for larger volumes (S4 Table). As a result, 2 aL synapses where characterized by small noisiness (median of 12%), but in the larger ones the level of noise became significant (median of 50% and 84% for 10 aL and 20 aL, respectively). In general, the noisiness of the system is high, i.e. highly variable reaction outcomes can be expected from one synapse to another.

### The Cu(II)—induced Aβ dimerization in the excited state of the synapse

**Fig 10:**
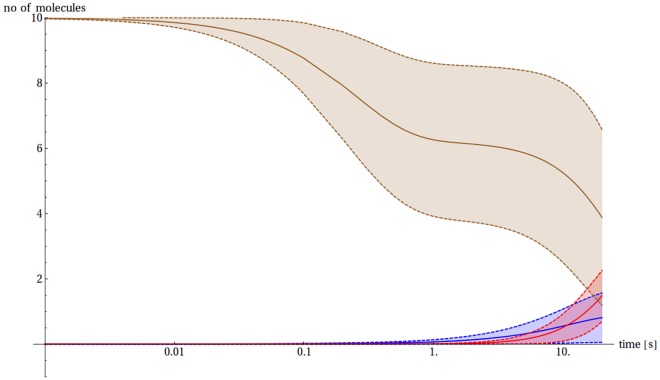
The sample evolution of the model in the excited state. The expected number of Aβ monomers (blue), CuAβ I (brown) and CuAβ_2_ (red) according to the model of Branch et al. [27] together with their standard deviations in the case where 10 Aβ molecules are present initially in the 5 aL volume after the release of 50 Cu(II) ions. The time scale is logarithmic.

In the next step we reconstructed the situation of release of a large quantity of Cu(II) ions upon the synaptic excitation. The number of Cu(II) ions in calculations ranged from 50 to 500, which corresponded to physiological concentrations reported, while taking actual synaptic cleft volumes into account. In this case the initial overflow of the system with Cu(II) ions quickly becomes neutralized by their steady efflux due to diffusion. The diffusion coefficient was estimated for the known physical parameters of the synaptic cleft fluid from the Stokes-Einstein equation as 3.45 × 10^−10^ m^2^s^-1^. This value is in a good agreement with experimental values recorded in low viscosity media (see S2 Text for details). The time window when Cu(II) ions are present in high numbers in the system is narrow, on the order of 100 ms, but it is sufficient for the CuAβ I and CuAβ II formation. Consequently, the Cu(II) ions stay in the cleft sufficiently long to totally saturate the pool of Aβ present in the system (Fig 10).

**Fig 11:**
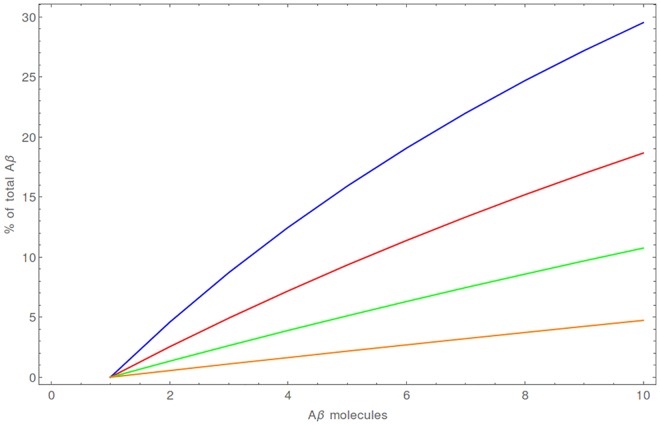
The average fraction of total Aβ present as the CuAβ2 complex[%] for the 5aL synaptic cleft after a single large Cu(II) release. Results are presented for release of 50 (blue), 100 (red), 200 (green) and 500 (orange) Cu(II) ions. The results for CuAβ_2_ in other volumes are presented in S6 Table.

The level of saturation exceeds 99% in all situations in the 4 ms timescale (S5 Table). The level of formation of CuAβ_2_ in a longer time perspective is in general low, but strongly depends on conditions. Predictably, the most favorable cases are when the smallest number of ions is released (50 in our calculations), the volume is small and the total number of Aβ molecules is high. In the extreme case (2 aL, 50 ions released), on average up to 51% of Aβ is present as dimers after 20 s. In general, in case of 50 released ions the values range from 10 to 20% (for 10 aL and 5 aL correspondingly; S6 Table). When higher Cu(II) amounts are present, the Aβ peptides become caught in CuAβ I and CuAβ II complexes and the fraction of Aβ present as CuAβ_2_ is on the level of just a few percent (Fig 11). In general, these calculations indicate that a single synaptic excitation tends to prevent the formation of CuAβ_2_, but not without exceptions.

The initial system overload with Cu(II) ions determines its behavior. Such situation drastically eliminates any possible noise, because initially all of the Aβ molecules bind the Cu(II) ions. The time on the level of seconds is required until any CuAβ complex will dissociate and a CuAβ_2_ complex may form. Only after this initial interval the noisiness of the system starts to become significant. The RSD values for CuAβ_2_ are relatively high in the 20 seconds timescale. The lowest RSD value is 0.34 for the case, when the potential dimer formation is strongest (10 Aβ molecules and 50 Cu(II) ions released into the 2 aL volume). In general the RSD is high, about 1.36 to 2.93 for cases when the CuAβ_2_ presence is noticeable (over one molecule on average), with the median of 2.52 for all cases. Due to the diffusion the starting number of Cu(II) ions quickly decreases to less than 10 after 2 s and typically less than one after 20 s (Figure A in S2 Text). In 20 aL and in cases when 10 Aβ molecules are present in the system the level of noise is therefore comparable to the noise observed in the resting state.

### The Cu(II)-induced Aβ dimerization after strong synaptic stimulation

**Fig 12:**
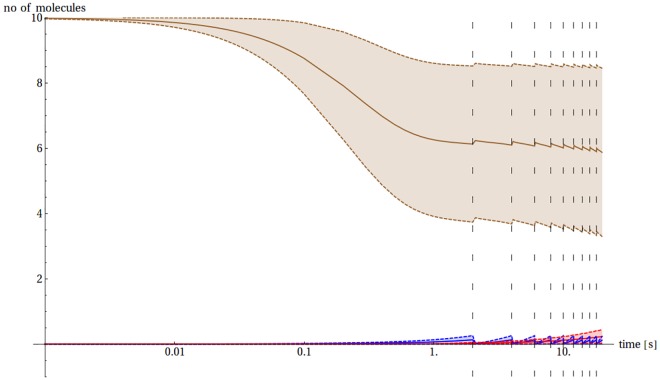
The sample evolution of the model after chronic stimulation. The expected number of Aβ monomers (blue), CuAβ I (brown) and CuAβ_2_ (red) according to the model of Branch et al. [27] together with their standard deviation in the case when 10 Aβ molecules are present initially in the 5 aL volume after a series of regular releases of 100 Cu(II) ions every 2 s. The moments of Cu(II) release are marked by the dashed lines. The time scale is logarithmic. Results for the other mode of strong stimulation are analogous (S10 Table).

Finally we aimed to model conditions present in the synaptic cleft during an intense stimulation. Two specific modes of such stimulation were considered. In one a chronic stimulation was applied over the interval of 20 s, with excitation, and consequently Cu(II) release occurring every 2 s (Fig 12). In another the strong excitation was composed of a train of impulses which led to a series of ten outbursts of Cu(II) ions in 4 ms intervals. In both cases the continuous release resulted in a large pool of Cu(II) ions remaining in the synaptic cleft for an extended period of time.

**Fig 13:**
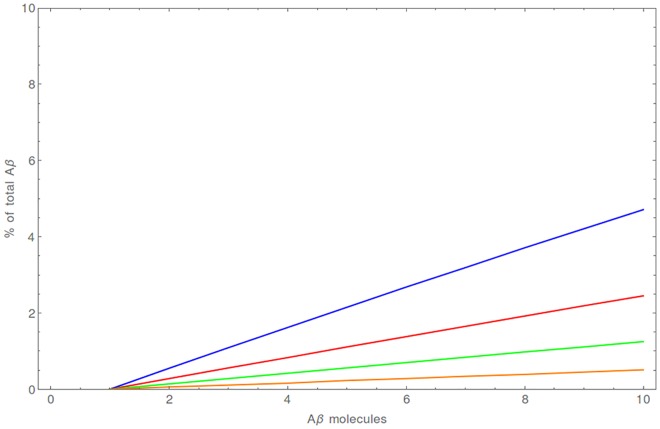
The average fraction of total Aβ as CuAβ_2_ complex [%] for the 5 aL volume after a train of Cu(II) releases. Results are presented for the release of 50 (blue), 100 (red), 200 (green) and 500 (orange) Cu(II) ions. The results for CuAβ_2_ in other volumes are presented in S8 Table.

Consequently the second mode becomes an extreme case of the first one. Aβ molecules become completely saturated with Cu(II) ions and the presence of CuAβ_2_ is negligible (~ 1% in the most favorable cases). The immediate formation of CuAβ I complexes determines the system, as there is not enough time (in repetitive stimulation, S8 Table) or there is too much Cu(II) available (in the strong stimulation mode) to form the dimers (Fig 13, S10 Table). In consequence, the average extent of CuAβ_2_ formation is negligible, making the respective RSD values meaningless. In other words in this model the system behaves in a deterministic way, i. e. is fully predictable and can be described correctly using the formalism of rate and equilibrium constants.

## Discussion

The synaptic cleft is a key site of release of Aβ peptides as a result of proteolysis of the APP protein. Our analysis demonstrated that the specific conditions present in this very small extracellular compartment, such as co-localization and proximity of potential reagents in the constrained space, result in a behavior of reagents that is different from that observed in bulk experiments (test tube scale). The main difference arises from the fact that in extremely small volumes of synaptic clefts (2–20 aL) concentrations, considered to be physiologically relevant for both Aβ peptides and Cu(II) ions correspond to just a few molecules/ions (for μM concentrations) or no molecules at all (for nM concentrations). As shown in Fig 3, the 1 μM concentration in 20 aL translates to 12 molecules, and one molecule in 2 aL constitutes a 833 nM concentration.

**Fig 14:**
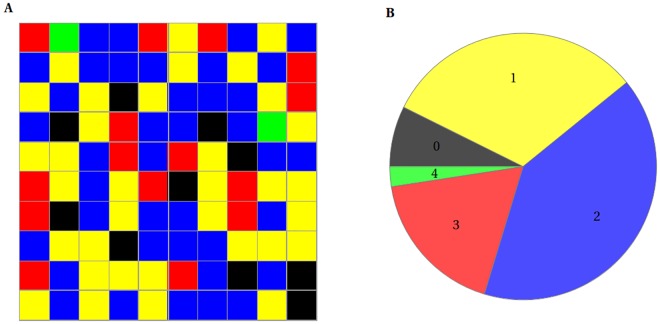
The dispersion in the possible reaction outcomes and its distribution. (A) Sample outcomes of 100 simulations in the time scale of 20 s in case of 10 Aβ and 5 Cu(II) in 20 aL. Color codes the number of CuAβ_2_ complexes (0—black, 1—yellow, 2—blue, 3—red, 4—green). The results are ordered by the number of a trial calculation. (B) Distribution of numbers of CuAβ_2_ complexes.

As explained above and illustrated in Fig 3, in such small volumes the generally accepted concept of concentration as a measure of presence of chemical species breaks down. The stochastic description must be used for chemical reactions, as the presence of just a few molecules of interacting species stipulates the probabilistic nature of their interactions. A high intrinsic noise (randomness of the reaction outcome) produced by these circumstances leads to sporadic occurrence of highly unlikely events, which can alter the evolution of the system in time. Consequently, using concentrations of Cu(II) ions and Aβ peptides alone in the kinetic analysis, one cannot predict the fate of the evolving system. The probabilistic factor must be taken into account. This approach can be explained using the results presented in Fig 14 as an example. We performed 100 independent calculations of the outcome of Cu(II)—induced dimerization after 20 s, for the initial 10 Aβ molecules and 5 Cu(II) ions in the 20 aL volume. The observed number of CuAβ_2_ complexes varied from 0 to 4 in individual calculations, with each of these possibilities having substantial probabilities of occurrence. This spread of results can be therefore described as a distribution of the potential outcomes (Fig 14B) defining the level of noise.

The other factor contributing to the variability of the Cu(II)—Aβ system is the size of the synaptic cleft, which can vary from 2 aL to 20 aL. As a consequence, the same ensemble of molecules may yield different quantities of reaction products, depending on the volume. In our work we report the average outcomes (correlated with the results from macroscopic experiments) in various conditions, in addition to the approximate level of noise.

Experimental studies on the aggregation of Aβ peptides established a tentative consensus regarding the particular neurotoxicity of small oligomers, including dimers [6,71]. It is also clear that the aggregation pathway is defined at the very first steps of the process and is determined by the initial conditions [16,20]. The role of Cu(II) ions as factors accelerating the Aβ aggregation has also been studied thoroughly by several research groups. We focus on the pathway to CuAβ_2_ complexes, being dimers par excellence and compare it with the apopetide dimerization. Our calculations are based on the most recent self-consistent set of kinetic data published by Branch et al. [27]. Their model of interactions is sufficiently complete to provide a basis for qualitative description of the systematic noise, which arises from the small number of interacting Aβ molecules and Cu(II) ions and therefore is an inherent part of all possible mechanisms. One has to stress, however, that our approach is technically universal and can adopt any new or expanded kinetic models of interactions. Our analysis can be treated as extended discussion of results of Branch et al., or as a Gedankenexperiment in which we try to look at consequences of a certain set of experimental data. Its most obvious limitations are listed in the last paragraph of the discussion.

**Fig 15:**
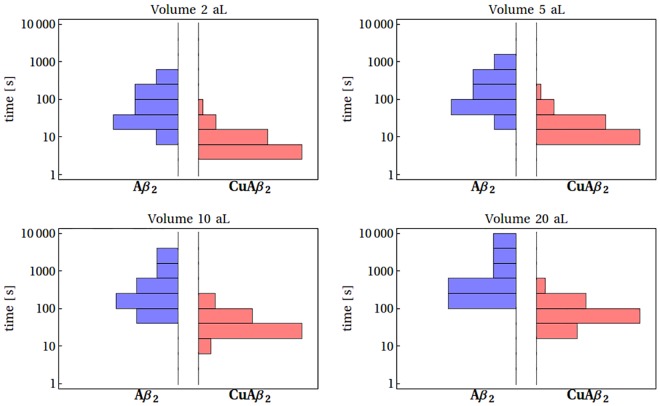
The histograms of the time required for the formation of Aβ_2_ (blue) and CuAβ_2_ (red) dimers.

The first important result of our analysis is that the size of synaptic cleft alone forces the Aβ peptides to aggregate spontaneously. The concentration of 2 μM, which is sufficient for the Aβ42 peptide to aggregate in an isolated system, can be achieved by merely three Aβ42 molecules in the smallest (2 aL) synaptic clefts, and 24 molecules in the largest ones (20 aL). The unassisted Aβ aggregation is, however, slow. The time required for the formation of Aβ dimer (~3 min) is an order of magnitude longer than that required for the corresponding CuAβ_2_ dimer to form (~ 20 s), not to mention the formation of monomeric CuAβ I complexes, which is practically immediate (Fig 15).

The dimerization time depends strongly on concentrations and the volume of the cleft, but in every case it is significantly longer than the formation of Cu(II) complexes. Taking into account the limited synaptic volume and rates of neuronal firing, associated with the regular release of a variety of factors interacting with Aβ peptides, including Zn(II) and Cu(II) ions, one can risk an opinion that the aggregation of Aβ peptide alone is too slow to be significant even in the confinement of the synaptic cleft. One can therefore speculate that the metal free Aβ aggregation may only occur outside the active synaptic cleft.

**Fig 16:**
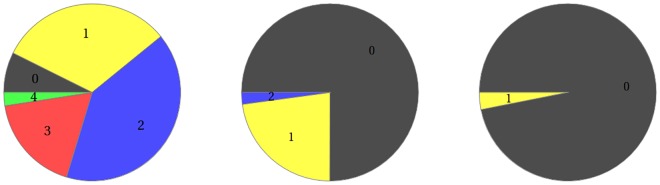
The distributions of CuAβ_2_ complex in the resting state, after single and chronic stimulation. Presented distributions describe the case of 10 Aβ molecules in 20 aL: left—in resting state with 5 Cu(II), middle—upon a single release of 100 Cu(II), right—upon a train of releases of 100 Cu(II) each.

We focused on the effect of Cu(II) ions due to their known association with the Aβ toxicity [72]. Results of our analysis are consistent with prior experimental papers on the role of Cu(II) ions in Aβ oligomerization and show that their presence drastically affects the propensity of the system for dimerization. The Cu(II)/Aβ ratio is the most influential factor here, yielding a very sharp dependence of the probable outcome of reactions. Whenever the number of Cu(II) ions is equal to or higher than that of Aβ molecules, the CuAβ I complexes are formed nearly immediately (within milliseconds). The Cu(II) availability is the only limiting factor here. This situation leads to a specific pathway culminating in amorphous aggregates, which are thought to be generally non-toxic [37,73]. In the alternative case of Aβ excess, the CuAβ_2_ complex can also be formed. The formation of CuAβ_2_ is most efficient when the Cu(II):Aβ ratio is exactly 1:2, that is under the sub-equimolar conditions studied by Pedersen and Smith [20,74]. This would explain the observed differences in aggregation pathways and strengthen the hypothesis that the formation of CuAβ_2_ promotes formation of toxic forms of Aβ [31,37]. The activity of the synapse is crucial in this context. The release of relatively high numbers of Cu(II) ions immediately changes the balance of the process, shifting it towards CuAβ I and CuAβ II complexes. This effect is not neglected by a high rate of clearance of Cu(II) from the cleft. Due to a relatively high affinity of Aβ peptides for Cu(II) ions, a nearly total saturation of the peptide takes place. Rephrasing, Aβ-bound Cu(II) ions remain in the system and a situation similar to that of the initial high Cu(II) excess can be observed. A series of impulses and consecutive releases of Cu(II) redirect the system towards the “Cu(II) excess” pathway with a simultaneous elimination of randomness of the reaction outcome (noise). Reduction of the noise is illustrated by narrowing down of the outcome distribution, which results from the rapid drop in the probability of CuAβ_2_ appearance (Fig 16). A high activity of the synapse is therefore redirecting the Aβ chemistry from aggregation towards the diffusible monomeric Cu(II) complex formation. We cannot state whether this effect decreases cytotoxicity or merely redirects is towards the oxidative pathway. Anyway this finding correlates the high synaptic activity with the decrease of the level of Aβ aggregation, which albeit simplistically, agrees with the known positive correlation of mental activity and resistance to Alzheimer’s disease.

According to our results, the main feature of Cu(II) dependent Aβ dimerization inside the synaptic cleft is its probabilistic nature. Small numbers of interacting molecules result in a wide range of possible reaction outcomes. Moreover, rare events have to be taken into account. When the system is not overloaded with Cu(II) ions, it is characterized by a high noise (Fig 16) which can alter the macroscopic evolution of the Cu(II)—Aβ system in a similar way as the stochastic factor controls the nucleation of Aβ_1–40_ peptide [75]. If the pathology of AD arises from a spontaneous formation of toxic and stable dimers, then the occurrence of the disease would have a stochastic nature, time (i.e. age) being necessarily a major risk factor. Moreover, the Cu(II) targeting therapies could be extremely difficult. A total depletion of synaptic Cu(II) is nearly impossible (and would be extremely harmful) and our results suggest that a partial Cu(II) depletion might actually accelerate rather than eliminate the neurotoxic Aβ dimer formation.

The level of noise varies also between synapses. Volumes of synaptic clefts are smaller in small synapses and are consequently characterized by more efficient interactions, due to the higher average proximity between the ligands (Fig 9). As such, their noisiness is lower when compared with the larger ones. On the other hand the size of the cleft corresponds to the size of dendritic spines. The mature and more stable spines are dominant in adult people and are characterized by larger sizes [42,76]. Our model suggests that such stabilization of dendritic spines distribution may enhance the probability of rare events, including the formation of CuAβ_2_ complexes.

Summarizing, in a properly functioning synaptic cleft, the accessibility of Cu(II) ions for Aβ binding should drive the aggregation towards the non toxic pathway, but the probabilistic nature of the reactions, especially in inactive synapses may bring the whole process on potentially more dangerous trails.

As already stated above, the kinetic model of Cu(II)-dependent Aβ peptide aggregation adopted and discussed above is of course very simplistic, largely by necessity. While the presence of multiple factors affecting the availability of Aβ peptides and Cu(II) ions is known, the quantities and kinetic properties of these factors (e.g. Cu(II) binding proteins) remain to be established. Thus, they could not be presently included in our model. Another important feature is the multiplicity of Aβ peptide forms. In this respect, we would like to mention the Aβ_4–42_ peptide, which is highly abundant in the brain [77–80]. We recently demonstrated this peptide to be a much stronger Cu(II) chelator than the Aβ_1–40_ or Aβ_1–42_ peptides (by a factor of 3000), and a likely physiological target for Cu(II) ions [81,82]. Its presence in the synaptic cleft may prevent Cu(II) interactions with the Aβ peptides considered in the kinetic model. The Aβ_4–42_ peptide also yields apparently toxic aggregates [83], but the effect of Cu(II) ions on their formation and properties remains to be established. One way of further development of our model will be to include Cu(II) binding properties of such potentially important molecules as soon as appropriate kinetic data become available.

## Supporting Information

S1 Table Dimerization without Cu(II). Average fraction of total Aβ bound as a Aβ_2_ complex [%].
(PDF)

S2 Table Resting state. Average fraction of total Aβ bound as a CuAβ I conformer [%] after 4ms.
(PDF)

S3 Table Resting state. Average fraction of total Aβ bound as a CuAβ_2_ complex [%] after 20 s.
(PDF)

S4 Table Resting state. RSD of CuAβ_2_ complex after 20 s.
(PDF)

S5 Table Excited state. Average fraction of total Aβ bound as a CuAβ I conformer [%] after 4ms.
(PDF)

S6 Table Excited state. Average fraction of total Aβ bound as a CuAβ_2_ complex [%] after 20 s.
(PDF)

S7 Table Excited state. RSD of CuAβ_2_ complex after 20 s.
(PDF)

S8 Table Regular long excitation. Average fraction of total Aβ bound as a CuAβ_2_ complex [%] after 20 s.
(PDF)

S9 Table Regular long excitation. RSD of CuAβ_2_ complex after 20 s.
(PDF)

S10 Table Short strong excitation. Average fraction of total Aβ bound as a CuAβ_2_ complex [%] after 20 s.
(PDF)

S11 Table Short strong excitation. RSD of CuAβ_2_ complex after 20 s.
(PDF)

S1 Text Chemical Master Equation. (PDF)

S2 Text Diffusion. (PDF)

S3 Text One molecule noise. (PDF)
