# Supplementary material for: Numerical Simulations Reveal Randomness of Cu(II) Induced Aβ Peptide Dimerization under Conditions Present in Glutamatergic Synapses
Source: PLoS One. 2017 Jan 26;12(1):e0170749. doi: 10.1371/journal.pone.0170749 (PMC5268396; doi:10.1371/journal.pone.0170749)
Supplement: S1 Table — Average fraction of total Aβ bound as a Aβ2 complex [%]. (PDF) [file pone.0170749.s001.pdf]

S1 Table. Dimerization without Cu(II). Average fraction of total Aβ bound as a $A\beta_2$ complex [%].

In all of the results the following color coding was applied: magenta – 2 aL, blue - 5 aL, red – 10 aL, green – 20 aL, grey – results not reliable due to small average number of molecules (i.e. <1)

Aβ	20s	5min	RSD 5min
1	0.	0.	NA
2	0.895963	16.473	1.74743
3	1.77592	27.8168	1.33395
4	2.64029	36.3	1.08074
5	3.4895	42.9218	0.906136
6	4.32394	48.2562	0.778644
7	5.144	52.6541	0.681515
8	5.95004	56.3479	0.605199
9	6.74242	59.4969	0.543737
10	7.5215	62.2153	0.493247

Aβ	20s	5min	RSD 5min
1	0.	0.	NA
2	0.359353	5.25679	1.89486
3	0.716127	9.97059	1.70088
4	1.07035	14.2241	1.53975
5	1.42204	18.0834	1.40403
6	1.77124	21.6023	1.28837
7	2.11797	24.825	1.18879
8	2.46226	27.788	1.10228
9	2.80412	30.5222	1.02655
10	3.14359	33.0535	0.959762

Aβ	20s	5min	RSD 5min
1	0.	0.	NA
2	0.179838	2.66388	1.94672
3	0.35903	5.18709	1.84439
4	0.537579	7.58068	1.75098
5	0.715487	9.85454	1.66546
6	0.892761	12.0176	1.58693
7	1.0694	14.0778	1.51462
8	1.24541	16.0425	1.44787
9	1.4208	17.9183	1.38609
10	1.59556	19.711	1.32879

Aβ	20s	5min	RSD 5min
1	0.	0.	NA
2	0.0899595	1.34093	1.97318
3	0.179757	2.64606	1.92062
4	0.269394	3.91681	1.87038
5	0.358869	5.15454	1.82234
6	0.448184	6.36053	1.77636
7	0.537339	7.536	1.73232
8	0.626334	8.68209	1.69011
9	0.71517	9.79991	1.64963
10	0.803847	10.8905	1.61078
