# Supplementary material for: Numerical Simulations Reveal Randomness of Cu(II) Induced Aβ Peptide Dimerization under Conditions Present in Glutamatergic Synapses
Source: PLoS One. 2017 Jan 26;12(1):e0170749. doi: 10.1371/journal.pone.0170749 (PMC5268396; doi:10.1371/journal.pone.0170749)
Supplement: S2 Table — Average fraction of total Aβ bound as a CuAβ I conformer [%] after 4ms. (PDF) [file pone.0170749.s002.pdf]

S2 Table. Resting state. Average fraction of total Aβ bound as a CuAβ I conformer [%] after 4ms.

Aβ\Cu	1	2	3	4	5	6	7	8	9	10
1	80.7116	95.9283	98.7877	99.3191	99.4138	99.4277	99.4275	99.4252	99.423	99.4212
2	47.9524	86.8869	96.7829	98.9015	99.3265	99.4073	99.4207	99.4215	99.4203	99.4189
3	32.9115	64.5065	89.9797	97.2923	98.9753	99.3305	99.4016	99.4146	99.4163	99.4158
4	24.8087	49.4247	72.9524	91.8363	97.6297	99.0266	99.3325	99.3964	99.4092	99.4116
5	19.8594	39.6971	59.3544	78.0864	93.0739	97.8693	99.0641	99.3334	99.3917	99.4044
6	16.5465	33.0971	49.6238	65.9842	81.5402	93.9575	98.0479	99.0924	99.3334	99.3874
7	14.178	28.3635	42.5513	56.7146	70.7248	84.0236	94.6198	98.186	99.1144	99.333
8	12.4014	24.8101	37.225	49.6409	62.0327	74.2829	85.8956	95.1345	98.2958	99.132
9	11.0196	22.0458	33.0785	44.1164	55.1544	66.1688	77.052	87.3572	95.5459	98.3852
10	9.91413	19.8344	29.7607	39.6925	49.6288	59.5646	69.4775	79.2681	88.5301	95.8822

Aβ\Cu	1	2	3	4	5	6	7	8	9	10
1	48.4282	73.2814	86.0335	92.5746	95.9283	97.6465	98.5258	98.9748	99.2034	99.3191
2	36.6377	63.135	79.3614	88.6104	93.6884	96.4162	97.8613	98.6196	99.0145	99.2188
3	28.6728	52.9032	71.1457	83.1233	90.3478	94.4864	96.7816	98.0275	98.694	99.0465
4	23.1372	44.2973	62.3371	76.2203	85.6852	91.5988	95.0861	97.0653	98.1599	98.7543
5	19.1781	37.4648	54.199	68.5422	79.7327	87.5438	92.5426	95.553	97.2919	98.2677
6	16.2661	32.1261	47.2299	61.0549	72.9469	82.3115	88.9543	93.2799	95.9267	97.4769
7	14.0662	27.9461	41.4615	54.3196	66.0901	76.24	84.2865	90.0616	93.8719	96.2326
8	12.3624	24.6393	36.7416	48.5137	59.7039	69.9476	78.7972	85.8481	90.9539	94.3574
9	11.0128	21.9865	32.8772	43.6044	54.03	63.9334	72.9986	80.8411	87.1141	91.6884
10	9.92177	19.8261	29.6914	39.4768	49.109	58.4635	67.3443	75.4728	82.5127	88.1612

Aβ\Cu	1	2	3	4	5	6	7	8	9	10
1	28.215	48.4282	62.9085	73.2814	80.7116	86.0335	89.8451	92.5746	94.5291	95.9283
2	24.2132	43.2346	57.8256	68.8368	77.0503	83.125	87.5892	90.8539	93.2322	94.9596
3	20.9679	38.5489	52.8663	64.2357	73.0828	79.8579	84.9814	88.8179	91.6684	93.7731
4	18.3181	34.4156	48.1749	59.6198	68.9058	76.2801	82.0325	86.4543	89.8131	92.3398
5	16.1396	30.8163	43.8461	55.1224	64.6345	72.4667	78.7772	83.7675	87.6518	90.6356
6	14.3358	27.7036	39.9239	50.8492	60.3864	68.512	75.2745	80.7827	85.1845	88.6446
7	12.8315	25.02	36.4143	46.8697	56.2647	64.5183	71.6028	77.5469	82.4294	86.3638
8	11.5681	22.7073	33.2993	43.2196	52.3479	60.5819	67.8507	74.1257	79.4244	83.8061
9	10.4992	20.7115	30.5477	39.908	48.6869	56.7823	64.1063	70.5965	76.225	81.0022
10	9.5886	18.9846	28.1227	36.9257	45.3076	53.1774	60.4468	67.0391	72.8989	77.9994

Aβ\Cu	1	2	3	4	5	6	7	8	9	10
1	15.281	28.215	39.1624	48.4282	56.2707	62.9085	68.5265	73.2814	77.3057	80.7116
2	14.1072	26.3294	36.8873	45.9847	53.807	60.5204	66.2732	71.1959	75.4034	78.9958
3	13.0537	24.5911	34.7405	43.6314	51.3912	58.1415	63.9968	69.0628	73.436	77.2036
4	12.1064	22.9915	32.7229	41.3774	49.0373	55.7876	61.7129	66.8956	71.4145	75.3434
5	11.2533	21.5216	30.8335	39.2291	46.7572	53.4731	59.4363	64.7083	69.3509	73.4246
6	10.4837	20.1719	29.0691	37.1903	44.56	51.2108	57.1813	62.515	67.258	71.4581
7	9.78834	18.9333	27.4251	35.2624	42.4528	49.0116	54.961	60.3292	65.1487	69.4552
8	9.15888	17.7968	25.896	33.4451	40.4403	46.8843	52.787	58.1638	63.0358	67.428
9	8.5881	16.7539	24.4757	31.7366	38.5252	44.8359	50.6691	56.0307	60.9319	65.3886
10	8.06963	15.7965	23.1577	30.1336	36.7085	42.8713	48.6157	53.9402	58.8485	63.3486
