# Supplementary material for: Numerical Simulations Reveal Randomness of Cu(II) Induced Aβ Peptide Dimerization under Conditions Present in Glutamatergic Synapses
Source: PLoS One. 2017 Jan 26;12(1):e0170749. doi: 10.1371/journal.pone.0170749 (PMC5268396; doi:10.1371/journal.pone.0170749)
Supplement: S3 Table — Average fraction of total Aβ bound as a CuAβ2 complex [%] after 20 s. (PDF) [file pone.0170749.s003.pdf]

S3 Table. Resting state. Average fraction of total Aβ bound as a $CuA\beta_2$ complex [%] after 20 s.

Aβ\Cu	1	2	3	4	5	6	7	8	9	10
1	0	0	0	0	0	0	0	0	0	0
2	64.6825	0.273076	0.136846	0.0912992	0.0685003	0.0548126	0.0456841	0.039162	0.0342696	0.0304638
3	58.2196	58.3624	0.537688	0.271037	0.181299	0.136229	0.109114	0.0910049	0.0780514	0.0683284
4	47.6898	75.8671	47.8934	0.796705	0.403328	0.270307	0.203342	0.162994	0.136018	0.116709
5	39.3148	72.3274	72.4578	39.6122	1.05101	0.534077	0.358496	0.269936	0.216513	0.180764
6	33.1181	64.1875	81.6237	64.3607	33.5566	1.30084	0.66346	0.445972	0.336077	0.269713
7	28.5007	56.3227	79.0936	79.2068	56.5471	29.1143	1.54617	0.79157	0.532799	0.401807
8	24.9759	49.7217	72.6644	85.1454	72.8145	50.0391	25.7846	1.78686	0.918443	0.619016
9	22.2137	44.3476	65.8426	83.1377	83.2365	66.0267	44.7963	23.2286	2.0227	1.04409
10	19.9969	39.9654	59.7063	77.8559	87.5264	77.988	59.9579	40.5722	21.2236	2.25348

Aβ\Cu	1	2	3	4	5	6	7	8	9	10
1	0	0	0	0	0	0	0	0	0	0
2	34.1522	0.271892	0.136546	0.0911694	0.0684249	0.0547643	0.0456505	0.0391383	0.0342507	0.0304488
3	37.6998	37.8001	0.534922	0.270371	0.181009	0.136068	0.109012	0.0909346	0.078001	0.0682891
4	35.6291	49.7946	35.8364	0.791958	0.402227	0.269838	0.203085	0.162833	0.135908	0.116628
5	32.3757	52.2528	52.3518	32.6947	1.0439	0.532472	0.357826	0.269574	0.216287	0.18061
6	29.1089	50.8318	59.2496	51.0167	29.551	1.29101	0.661285	0.445076	0.335597	0.269417
7	26.1576	47.8977	60.9217	61.0127	48.1611	26.7376	1.53328	0.788758	0.531656	0.401201
8	23.5883	44.5069	59.8029	65.6418	59.9697	44.852	24.3213	1.77061	0.914934	0.617602
9	21.3813	41.1342	57.2905	66.8143	66.8972	57.5221	41.5719	22.2806	2.00282	1.03982
10	19.4914	37.9813	54.18	65.8562	70.2706	66.0079	54.4742	38.5267	20.568	2.22974

Aβ\Cu	1	2	3	4	5	6	7	8	9	10
1	0	0	0	0	0	0	0	0	0	0
2	18.8512	0.269945	0.13605	0.0909437	0.0682997	0.054684	0.0455947	0.0390963	0.0342192	0.0304247
3	22.7581	22.8261	0.530388	0.269269	0.180529	0.135801	0.108843	0.0908176	0.0779155	0.0682222
4	23.2578	31.4608	23.4222	0.784197	0.400408	0.269061	0.202659	0.162566	0.135724	0.116495
5	22.6167	34.761	34.828	22.8906	1.0323	0.529823	0.356714	0.268972	0.215912	0.180355
6	21.5555	35.6158	40.5561	35.7542	21.9482	1.27501	0.657699	0.443593	0.334802	0.268925
7	20.3583	35.2496	43.1892	43.2518	35.4635	20.878	1.51236	0.784129	0.529763	0.400195
8	19.1496	34.2572	44.0331	47.4618	44.1575	34.5509	19.804	1.74431	0.909159	0.615264
9	17.986	32.9526	43.8124	49.5745	49.6329	43.9985	33.3317	18.7825	1.97073	1.03281
10	16.8922	31.5107	42.9552	50.3176	52.8981	50.432	43.204	31.9817	17.8376	2.19151

Aβ\Cu	1	2	3	4	5	6	7	8	9	10
1	0	0	0	0	0	0	0	0	0	0
2	9.89345	0.266153	0.135072	0.0905035	0.0680509	0.0545276	0.0454835	0.0390144	0.0341564	0.0303743
3	12.555	12.6129	0.521586	0.267098	0.179576	0.135271	0.108512	0.0905847	0.077745	0.0680938
4	13.4408	18.0087	13.5863	0.769198	0.396828	0.267523	0.201814	0.162033	0.135359	0.116229
5	13.65	20.6268	20.6798	13.8971	1.01	0.524621	0.354516	0.267777	0.215166	0.179847
6	13.5476	21.8932	24.7383	22.0052	13.9044	1.24438	0.650666	0.440662	0.333225	0.267948
7	13.2879	22.4214	27.0897	27.1369	22.5984	13.7599	1.47251	0.775068	0.526027	0.398203
8	12.9465	22.5172	28.4065	30.4197	28.5018	22.7642	13.538	1.69443	0.897878	0.610654
9	12.5637	22.3486	29.0633	32.4958	32.5385	29.2091	22.6699	13.2783	1.91015	1.01912
10	12.1626	22.0148	29.2841	33.7536	35.2854	33.8386	29.483	22.4145	13.0034	2.11966
