# Supplementary material for: Numerical Simulations Reveal Randomness of Cu(II) Induced Aβ Peptide Dimerization under Conditions Present in Glutamatergic Synapses
Source: PLoS One. 2017 Jan 26;12(1):e0170749. doi: 10.1371/journal.pone.0170749 (PMC5268396; doi:10.1371/journal.pone.0170749)
Supplement: S4 Table — RSD of CuAβ2 complex after 20 s. (PDF) [file pone.0170749.s004.pdf]

S4 Table. Resting state. RSD of $CuA\beta_2$ complex after 20 s

Aβ\Cu	1	2	3	4	5	6	7	8	9	10
1	NA	NA	NA	NA	NA	NA	NA	NA	NA	NA
2	0.739	19.11	27.014	33.08	38.195	42.701	46.775	50.522	54.01	57.285
3	0.381	0.377	11.09	15.651	19.15	22.099	24.698	27.047	29.208	31.22
4	0.22	0.35	0.219	7.864	11.094	13.568	15.653	17.49	19.15	20.678
5	0.132	0.221	0.219	0.147	6.1	8.608	10.526	12.142	13.565	14.851
6	0.081	0.137	0.226	0.135	0.131	4.983	7.036	8.605	9.925	11.087
7	0.05	0.085	0.156	0.155	0.088	0.147	4.21	5.951	7.279	8.396
8	0.031	0.053	0.101	0.166	0.099	0.071	0.173	3.642	5.156	6.308
9	0.02	0.033	0.064	0.121	0.12	0.065	0.074	0.201	3.208	4.547
10	0.012	0.021	0.04	0.08	0.131	0.079	0.05	0.086	0.228	2.864

Aβ\Cu	1	2	3	4	5	6	7	8	9	10
1	NA	NA	NA	NA	NA	NA	NA	NA	NA	NA
2	1.389	19.152	27.044	33.105	38.216	42.72	46.793	50.538	54.025	57.299
3	0.877	0.874	11.119	15.671	19.165	22.112	24.709	27.058	29.218	31.229
4	0.635	0.613	0.632	7.888	11.109	13.58	15.663	17.499	19.158	20.685
5	0.485	0.47	0.469	0.485	6.121	8.621	10.536	12.15	13.572	14.857
6	0.381	0.372	0.384	0.37	0.387	5.002	7.048	8.614	9.932	11.093
7	0.304	0.299	0.318	0.317	0.297	0.323	4.228	5.962	7.287	8.402
8	0.245	0.242	0.264	0.276	0.263	0.243	0.285	3.66	5.166	6.315
9	0.198	0.197	0.218	0.239	0.238	0.217	0.202	0.266	3.225	4.557
10	0.162	0.161	0.181	0.204	0.215	0.203	0.18	0.173	0.262	2.88

Aβ\Cu	1	2	3	4	5	6	7	8	9	10
1	NA	NA	NA	NA	NA	NA	NA	NA	NA	NA
2	2.075	19.221	27.093	33.145	38.251	42.751	46.821	50.565	54.049	57.322
3	1.389	1.386	11.167	15.703	19.191	22.134	24.729	27.075	29.234	31.243
4	1.072	0.952	1.068	7.927	11.135	13.6	15.68	17.513	19.171	20.697
5	0.877	0.749	0.748	0.873	6.156	8.643	10.553	12.164	13.583	14.867
6	0.739	0.622	0.599	0.62	0.738	5.035	7.068	8.628	9.944	11.103
7	0.635	0.531	0.504	0.504	0.529	0.639	4.259	5.98	7.3	8.413
8	0.553	0.461	0.436	0.43	0.435	0.459	0.564	3.69	5.183	6.328
9	0.485	0.404	0.382	0.377	0.376	0.381	0.404	0.507	3.253	4.573
10	0.429	0.357	0.339	0.334	0.333	0.333	0.338	0.36	0.465	2.908

Aβ\Cu	1	2	3	4	5	6	7	8	9	10
1	NA	NA	NA	NA	NA	NA	NA	NA	NA	NA
2	3.018	19.358	27.191	33.225	38.321	42.812	46.879	50.618	54.099	57.369
3	2.076	2.07	11.261	15.767	19.242	22.178	24.767	27.11	29.266	31.274
4	1.649	1.435	1.64	8.006	11.185	13.639	15.713	17.542	19.197	20.72
5	1.389	1.147	1.145	1.379	6.226	8.686	10.586	12.191	13.607	14.889
6	1.208	0.971	0.919	0.968	1.198	5.099	7.106	8.657	9.968	11.124
7	1.072	0.848	0.782	0.781	0.845	1.063	4.319	6.016	7.326	8.434
8	0.965	0.755	0.686	0.667	0.684	0.752	0.959	3.747	5.216	6.352
9	0.877	0.682	0.613	0.587	0.587	0.611	0.679	0.875	3.309	4.605
10	0.803	0.622	0.556	0.527	0.518	0.526	0.554	0.62	0.807	2.962
