# Supplementary material for: Numerical Simulations Reveal Randomness of Cu(II) Induced Aβ Peptide Dimerization under Conditions Present in Glutamatergic Synapses
Source: PLoS One. 2017 Jan 26;12(1):e0170749. doi: 10.1371/journal.pone.0170749 (PMC5268396; doi:10.1371/journal.pone.0170749)
Supplement: S5 Table — Average fraction of total Aβ bound as a CuAβ I conformer [%] after 4ms. (PDF) [file pone.0170749.s005.pdf]

S5 Table. Excited state. Average fraction of total Aβ bound as a CuAβ I conformer [%] after 4ms

Aβ\Cu	50	100	200	500
1	99.4055	99.4051	99.4049	99.4048
2	99.4051	99.4049	99.4048	99.4048
3	99.4047	99.4047	99.4047	99.4048
4	99.4043	99.4045	99.4046	99.4047
5	99.4039	99.4043	99.4045	99.4047
6	99.4035	99.4041	99.4044	99.4046
7	99.4031	99.4039	99.4043	99.4046
8	99.4027	99.4037	99.4042	99.4046
9	99.4023	99.4035	99.4041	99.4045
10	99.4019	99.4033	99.404	99.4045

Aβ\Cu	50	100	200	500
1	99.4119	99.4083	99.4065	99.4055
2	99.4115	99.4081	99.4064	99.4054
3	99.4111	99.4079	99.4063	99.4054
4	99.4107	99.4077	99.4062	99.4054
5	99.4103	99.4075	99.4061	99.4053
6	99.4099	99.4073	99.406	99.4053
7	99.4095	99.4071	99.4059	99.4052
8	99.4091	99.4069	99.4058	99.4052
9	99.4087	99.4067	99.4057	99.4052
10	99.4083	99.4065	99.4056	99.4051

Aβ\Cu	50	100	200	500
1	99.4211	99.4129	99.4088	99.4064
2	99.4207	99.4127	99.4087	99.4064
3	99.4203	99.4125	99.4086	99.4063
4	99.4199	99.4123	99.4085	99.4063
5	99.4195	99.4121	99.4084	99.4062
6	99.4191	99.4119	99.4083	99.4062
7	99.4187	99.4117	99.4082	99.4062
8	99.4183	99.4115	99.4081	99.4061
9	99.4179	99.4113	99.408	99.4061
10	99.4175	99.4111	99.4079	99.406

Aβ\Cu	50	100	200	500
1	99.4138	99.4212	99.413	99.4081
2	99.4134	99.421	99.4129	99.408
3	99.413	99.4208	99.4128	99.408
4	99.4126	99.4206	99.4127	99.4079
5	99.4122	99.4204	99.4126	99.4079
6	99.4118	99.4202	99.4125	99.4079
7	99.4114	99.42	99.4124	99.4078
8	99.411	99.4198	99.4123	99.4078
9	99.4106	99.4196	99.4122	99.4077
10	99.4102	99.4194	99.4121	99.4077
