# Supplementary material for: Numerical Simulations Reveal Randomness of Cu(II) Induced Aβ Peptide Dimerization under Conditions Present in Glutamatergic Synapses
Source: PLoS One. 2017 Jan 26;12(1):e0170749. doi: 10.1371/journal.pone.0170749 (PMC5268396; doi:10.1371/journal.pone.0170749)
Supplement: S6 Table — Average fraction of total Aβ bound as a CuAβ2 complex [%] after 20 s. (PDF) [file pone.0170749.s006.pdf]

S6 Table. Excited state. Average fraction of total Aβ bound as a $CuA\beta_2$ complex [%] after 20 s.

Aβ\Cu	50	100	200	500
1	0	0	0	0
2	10.9744	6.17136	3.2899	1.37039
3	19.5061	11.5473	6.34963	2.70035
4	26.3974	16.283	9.20394	3.99172
5	32.1085	20.4933	11.874	5.24626
6	36.9343	24.2659	14.3781	6.46559
7	41.0749	27.6691	16.7319	7.65124
8	44.6725	30.7572	18.9493	8.80467
9	47.8311	33.5739	21.0423	9.92721
10	50.6292	36.1551	23.0217	11.0202

Aβ\Cu	50	100	200	500
1	0	0	0	0
2	4.57279	2.52654	1.33264	0.551154
3	8.70931	4.91753	2.62717	1.09574
4	12.4727	7.18415	3.88528	1.63389
5	15.9138	9.33634	5.10855	2.16571
6	19.0745	11.383	6.29848	2.69133
7	21.9891	13.332	7.45649	3.21084
8	24.6866	15.1905	8.58388	3.72438
9	27.1914	16.965	9.68192	4.23203
10	29.5242	18.6613	10.7518	4.73391

Aβ\Cu	50	100	200	500
1	0	0	0	0
2	2.31913	1.27369	0.669413	0.276195
3	4.52493	2.51273	1.32918	0.550739
4	6.62588	3.71859	1.97952	0.82365
5	8.62961	4.89263	2.62064	1.09494
6	10.543	6.03616	3.25274	1.36462
7	12.3723	7.15039	3.87601	1.63272
8	14.1231	8.23649	4.49067	1.89923
9	15.8006	9.29556	5.09687	2.1642
10	17.4094	10.3286	5.69482	2.4276

Aβ\Cu	50	100	200	500
1	0	0	0	0
2	1.16876	0.639968	0.335746	0.138361
3	2.3086	1.27116	0.669061	0.276306
4	3.42061	1.89377	0.999973	0.413839
5	4.50584	2.50798	1.32851	0.550962
6	5.56528	3.11396	1.6547	0.687677
7	6.59987	3.71188	1.97856	0.823985
8	7.61052	4.30191	2.30013	0.959888
9	8.59807	4.88421	2.61942	1.09539
10	9.56333	5.45894	2.93647	1.23049
