# Supplementary material for: Numerical Simulations Reveal Randomness of Cu(II) Induced Aβ Peptide Dimerization under Conditions Present in Glutamatergic Synapses
Source: PLoS One. 2017 Jan 26;12(1):e0170749. doi: 10.1371/journal.pone.0170749 (PMC5268396; doi:10.1371/journal.pone.0170749)
Supplement: S7 Table — RSD of CuAβ2 complex after 20 s. (PDF) [file pone.0170749.s007.pdf]

S7 Table. Excited state. RSD of $CuA\beta_2$ complex after 20 s.

Aβ\Cu	50	100	200	500
1	NA	NA	NA	NA
2	3.02	4.03	5.51	8.54
3	1.85	2.4	3.24	4.97
4	1.45	1.81	2.37	3.56
5	1.28	1.52	1.92	2.82
6	1.18	1.35	1.66	2.36
7	1.13	1.26	1.49	2.06
8	1.09	1.19	1.38	1.84
9	1.07	1.15	1.3	1.69
10	1.06	1.12	1.24	1.57

Aβ\Cu	50	100	200	500
1	NA	NA	NA	NA
2	4.68	6.29	8.66	13.47
3	2.77	3.68	5.04	7.8
4	2.05	2.67	3.61	5.55
5	1.69	2.15	2.85	4.33
6	1.48	1.83	2.39	3.58
7	1.35	1.63	2.08	3.06
8	1.27	1.49	1.86	2.69
9	1.21	1.39	1.7	2.42
10	1.17	1.32	1.58	2.2

Aβ\Cu	50	100	200	500
1	NA	NA	NA	NA
2	6.57	8.86	12.22	19.03
3	3.84	5.15	7.08	11.
4	2.78	3.69	5.04	7.8
5	2.23	2.91	3.95	6.07
6	1.89	2.44	3.26	4.98
7	1.68	2.12	2.8	4.24
8	1.53	1.89	2.47	3.7
9	1.42	1.73	2.23	3.29
10	1.34	1.61	2.04	2.98

Aβ\Cu	50	100	200	500
1	NA	NA	NA	NA
2	9.25	12.5	17.26	26.88
3	5.37	7.24	9.98	15.53
4	3.85	5.15	7.08	11.
5	3.03	4.03	5.51	8.54
6	2.53	3.33	4.53	6.99
7	2.2	2.86	3.86	5.93
8	1.96	2.52	3.38	5.16
9	1.78	2.27	3.01	4.57
10	1.65	2.07	2.73	4.11
