# Supplementary material for: Numerical Simulations Reveal Randomness of Cu(II) Induced Aβ Peptide Dimerization under Conditions Present in Glutamatergic Synapses
Source: PLoS One. 2017 Jan 26;12(1):e0170749. doi: 10.1371/journal.pone.0170749 (PMC5268396; doi:10.1371/journal.pone.0170749)
Supplement: S8 Table — Average fraction of total Aβ bound as a CuAβ2 complex [%] after 20 s. (PDF) [file pone.0170749.s008.pdf]

S8 Table. Regular long excitation. Average fraction of total Aβ bound as a $CuAβ_2$ complex [%] after 20 s

Aβ\Cu	50	100	200	500
1	0.	0.	0.	0.
2	1.35906	0.693366	0.350259	0.140981
3	2.6785	1.37637	0.697868	0.281525
4	3.96008	2.04925	1.04286	0.421646
5	5.20549	2.71223	1.38525	0.561337
6	6.4163	3.36556	1.72509	0.700609
7	7.59399	4.00943	2.0624	0.839453
8	8.73996	4.64407	2.39721	0.97788
9	9.85553	5.26967	2.72955	1.11589
10	10.9419	5.88644	3.05944	1.25348

Aβ\Cu	50	100	200	500
1	0.	0.	0.	0.
2	0.548131	0.278947	0.140731	0.0565982
3	1.08979	0.556213	0.281031	0.11313
4	1.6251	0.831815	0.420907	0.169591
5	2.15417	1.10577	0.560358	0.22598
6	2.67712	1.37809	0.699387	0.282305
7	3.19406	1.64879	0.837994	0.338555
8	3.70508	1.91788	0.976184	0.39474
9	4.21031	2.18539	1.11396	0.450853
10	4.70983	2.45132	1.25132	0.506899

Aβ\Cu	50	100	200	500
1	0.	0.	0.	0.
2	0.275999	0.140334	0.0707705	0.028453
3	0.550355	0.280244	0.141426	0.0568885
4	0.823085	0.419728	0.211981	0.0853066
5	1.0942	0.558792	0.282421	0.113709
6	1.36372	0.697436	0.352756	0.142094
7	1.63166	0.835663	0.422984	0.170458
8	1.89803	0.973474	0.493105	0.198804
9	2.16285	1.11087	0.56312	0.227135
10	2.42612	1.24786	0.633026	0.255448

Aβ\Cu	50	100	200	500
1	0.	0.	0.	0.
2	0.139603	0.0709487	0.035767	0.0143785
3	0.278786	0.141783	0.0715066	0.0287525
4	0.41755	0.212512	0.10722	0.043122
5	0.555899	0.283133	0.142902	0.0574867
6	0.693833	0.353646	0.17856	0.0718475
7	0.831355	0.424051	0.214188	0.0862033
8	0.968466	0.494349	0.249789	0.100556
9	1.10517	0.564539	0.285363	0.114904
10	1.24146	0.634623	0.32091	0.129245
