# Supplementary material for: Numerical Simulations Reveal Randomness of Cu(II) Induced Aβ Peptide Dimerization under Conditions Present in Glutamatergic Synapses
Source: PLoS One. 2017 Jan 26;12(1):e0170749. doi: 10.1371/journal.pone.0170749 (PMC5268396; doi:10.1371/journal.pone.0170749)
Supplement: S9 Table — RSD of CuAβ2 complex after 20 s. (PDF) [file pone.0170749.s009.pdf]

S9 Table. Regular long excitation. RSD of $CuA\beta_2$ complex after 20 s

Aβ\Cu	50	100	200	500
1	NA	NA	NA	NA
2	8.57	12.	16.89	26.62
3	4.99	6.96	9.77	15.38
4	3.58	4.95	6.93	10.89
5	2.83	3.88	5.4	8.46
6	2.37	3.21	4.44	6.92
7	2.06	2.76	3.78	5.87
8	1.85	2.43	3.31	5.11
9	1.69	2.19	2.95	4.53
10	1.57	2.01	2.68	4.07

Aβ\Cu	50	100	200	500
1	NA	NA	NA	NA
2	13.5	18.93	26.64	42.02
3	7.82	10.94	15.4	24.26
4	5.56	7.76	10.9	17.17
5	4.34	6.04	8.46	13.31
6	3.58	4.96	6.93	10.88
7	3.07	4.22	5.88	9.21
8	2.7	3.68	5.11	7.99
9	2.42	3.28	4.53	7.06
10	2.21	2.96	4.07	6.33

Aβ\Cu	50	100	200	500
1	NA	NA	NA	NA
2	19.03	26.68	37.57	59.26
3	11.	15.42	21.7	34.22
4	7.8	10.92	15.36	24.2
5	6.07	8.47	11.91	18.76
6	4.98	6.94	9.74	15.32
7	4.24	5.89	8.24	12.96
8	3.7	5.12	7.15	11.23
9	3.29	4.54	6.33	9.92
10	2.98	4.08	5.67	8.88

Aβ\Cu	50	100	200	500
1	NA	NA	NA	NA
2	26.75	37.53	52.85	83.36
3	15.46	21.67	30.52	48.13
4	10.95	15.34	21.59	34.04
5	8.5	11.89	16.73	26.37
6	6.96	9.72	13.67	21.54
7	5.9	8.23	11.57	18.21
8	5.13	7.15	10.03	15.78
9	4.55	6.32	8.85	13.92
10	4.09	5.67	7.93	12.46
