# Supplementary material for: Numerical Simulations Reveal Randomness of Cu(II) Induced Aβ Peptide Dimerization under Conditions Present in Glutamatergic Synapses
Source: PLoS One. 2017 Jan 26;12(1):e0170749. doi: 10.1371/journal.pone.0170749 (PMC5268396; doi:10.1371/journal.pone.0170749)
Supplement: S10 Table — Average fraction of total Aβ bound as a CuAβ2 complex [%] after 20 s. (PDF) [file pone.0170749.s010.pdf]

S10 Table. Short strong excitation. Average fraction of total Aβ bound as a $CuA\beta_2$ complex [%] after 20 s.

Aβ\Cu	50	100	200	500
1	0.	0.	0.	0.
2	0.000544523	0.000272767	0.000136511	0.0000546356
3	0.00108903	0.00054553	0.000273021	0.000109271
4	0.00163352	0.00081829	0.000409531	0.000163905
5	0.002178	0.00109104	0.000546039	0.00021854
6	0.00272246	0.0013638	0.000682546	0.000273175
7	0.0032669	0.00163654	0.000819052	0.00032781
8	0.00381133	0.00190929	0.000955558	0.000382443
9	0.00435575	0.00218203	0.00109206	0.000437078
10	0.00490015	0.00245476	0.00122856	0.000491712

Aβ\Cu	50	100	200	500
1	0.	0.	0.	0.
2	0.000466218	0.000233383	0.00011676	0.0000467207
3	0.000932429	0.000466763	0.00023352	0.0000934414
4	0.00139863	0.000700143	0.00035028	0.000140162
5	0.00186483	0.000933519	0.000467038	0.000186882
6	0.00233102	0.00116689	0.000583797	0.000233603
7	0.0027972	0.00140027	0.000700555	0.000280323
8	0.00326338	0.00163364	0.000817312	0.000327044
9	0.00372955	0.00186701	0.000934069	0.000373764
10	0.00419571	0.00210038	0.00105083	0.000420484

Aβ\Cu	50	100	200	500
1	0.	0.	0.	0.
2	0.000440773	0.00022063	0.000110377	0.0000441654
3	0.00088154	0.000441259	0.000220753	0.0000883307
4	0.0013223	0.000661886	0.000331128	0.000132496
5	0.00176306	0.000882512	0.000441504	0.000176661
6	0.00220381	0.00110314	0.000551879	0.000220826
7	0.00264455	0.00132376	0.000662254	0.000264991
8	0.00308529	0.00154438	0.000772628	0.000309157
9	0.00352603	0.001765	0.000883002	0.000353321
10	0.00396675	0.00198562	0.000993376	0.000397486

Aβ\Cu	50	100	200	500
1	0.	0.	0.	0.
2	0.000428778	0.000214708	0.000107434	0.0000429927
3	0.000857552	0.000429415	0.000214868	0.0000859858
4	0.00128632	0.00064412	0.000322301	0.000128979
5	0.00171508	0.000858825	0.000429735	0.000171972
6	0.00214384	0.00107353	0.000537168	0.000214964
7	0.00257259	0.00128823	0.0006446	0.000257957
8	0.00300134	0.00150293	0.000752032	0.000300949
9	0.00343009	0.00171763	0.000859464	0.000343942
10	0.00385882	0.00193233	0.000966896	0.000386935
