# Supplementary material for: Numerical Simulations Reveal Randomness of Cu(II) Induced Aβ Peptide Dimerization under Conditions Present in Glutamatergic Synapses
Source: PLoS One. 2017 Jan 26;12(1):e0170749. doi: 10.1371/journal.pone.0170749 (PMC5268396; doi:10.1371/journal.pone.0170749)
Supplement: S11 Table — RSD of CuAβ2 complex after 20 s. (PDF) [file pone.0170749.s011.pdf]

S11 Table. Short strong excitation. RSD of $CuA\beta_2$ complex after 20 s.

Aβ\Cu	50	100	200	500
1	NA	NA	NA	NA
2	482.23	681.9	964.29	1524.64
3	278.42	393.7	556.74	880.25
4	196.87	278.39	393.67	622.43
5	152.5	215.64	304.94	482.13
6	124.51	176.07	248.98	393.66
7	105.24	148.81	210.43	332.7
8	91.14	128.87	182.24	288.13
9	80.38	113.65	160.72	254.11
10	71.89	101.66	143.75	227.28

Aβ\Cu	50	100	200	500
1	NA	NA	NA	NA
2	485.28	686.18	970.32	1534.13
3	280.18	396.17	560.22	885.74
4	198.12	280.13	396.14	626.32
5	153.46	216.99	306.85	485.14
6	125.3	177.17	250.54	396.12
7	105.9	149.74	211.75	334.78
8	91.71	129.68	183.38	289.93
9	80.89	114.37	161.72	255.69
10	72.35	102.29	144.65	228.7

Aβ\Cu	50	100	200	500
1	NA	NA	NA	NA
2	485.81	686.84	971.18	1535.44
3	280.49	396.55	560.71	886.49
4	198.33	280.4	396.49	626.84
5	153.63	217.2	307.12	485.55
6	125.44	177.34	250.76	396.45
7	106.02	149.88	211.93	335.06
8	91.81	129.8	183.54	290.17
9	80.97	114.48	161.87	255.91
10	72.43	102.39	144.78	228.89

Aβ\Cu	50	100	200	500
1	NA	NA	NA	NA
2	486.13	687.08	971.39	1535.62
3	280.67	396.69	560.83	886.59
4	198.46	280.5	396.57	626.91
5	153.73	217.28	307.18	485.61
6	125.52	177.41	250.81	396.5
7	106.09	149.94	211.98	335.1
8	91.88	129.85	183.58	290.21
9	81.03	114.52	161.9	255.94
10	72.47	102.43	144.81	228.92
