# Supplementary material for: Numerical Simulations Reveal Randomness of Cu(II) Induced Aβ Peptide Dimerization under Conditions Present in Glutamatergic Synapses
Source: PLoS One. 2017 Jan 26;12(1):e0170749. doi: 10.1371/journal.pone.0170749 (PMC5268396; doi:10.1371/journal.pone.0170749)
Supplement: S1 Text — (PDF) [file pone.0170749.s012.pdf]

S1 Text. Chemical Master Equation

Theory of Markov multi-state processes provides a description of chemical reactions system in terms of possible states in which system can be found and corresponding probabilities. Every state is determined by the numbers of molecules of every reagent. To every state i and every moment t one can assign the probability pi that the system S is in this particular state at time t:

$$p_i(t) = \mathbf{P}(S(t)=i)$$

Under mild assumptions every such time-dependent probability function satisfies the Markov property. Now, assuming that r is a reaction that transforms state i to state j, the probability of such transition in sufficiently small interval v is proportional to the rate of reaction r:

$$\mathbf{P}(S(t+\tau) = i | S(t)=j) = \tau\ (k_r\, j^r) + o(\tau^2)$$

where k_r is the rate of r reaction and j^r is the factor corresponding to the number of reacting molecules in state j. One can therefore derive a set of ordinary differential equations determining the evolution of pi functions in time, called Chemical Master Equation:

$$dp_i/dt = \lim_{\tau\text{->}0} (p_i(t+\tau) - p_i(\tau))/\tau = \sum_r r\ k_r\ (j_r^{\ r}\ p_j(t) - \ i^r\ p_i(t))$$

where j_r is such state j that r reaction transforms it into the state i.
