# Supplementary material for: Numerical Simulations Reveal Randomness of Cu(II) Induced Aβ Peptide Dimerization under Conditions Present in Glutamatergic Synapses
Source: PLoS One. 2017 Jan 26;12(1):e0170749. doi: 10.1371/journal.pone.0170749 (PMC5268396; doi:10.1371/journal.pone.0170749)
Supplement: S2 Text — (PDF) [file pone.0170749.s013.pdf]

S2 Text. Diffusion

Diffusion of the Cu(II) ions out of the synaptic cleft was estimated from the equations governing the Brownian Motion. The Brownian Motion satisfies:

$$E(x(t)^2) = 2Dt$$

where $E(x(t)^2)$ – is mean square displacement during the time t and D stands for the diffusion coefficient. This coefficient was estimated from the Stokes - Einstein relation:

$$D = k_B T/(6\pi\eta R)$$

where the η– viscosity, k_B – Boltzmann constant, T – temperature and R is radius of spherical particle. The viscosity of the fluid was assumed to be 3 (1) higher than water, and the radius of Cu(II) ion included the hydratation sphere and was equal to 310 pm.

Therefore the estimation of diffusion coefficient was:

$$D = 3.45\times10^{-6}\ cm^2/s.$$

This estimation is reliable, when compared to experimental and theoretical estimations for Aβ monomer (2,3):

$$D_{A\beta} = 0.6 - 2\times10^{-6}\ cm^2/s$$

Synaptic cleft was treated as round and very flat cylinder, in which the Brownian movement is collapsed to two dimensions (i.e. the D was rescaled by the factor of $\sqrt{2/3}$). The radius of the cleft was corelated with the assumed volume and corresponded to volumes of 2, 5, 10 and 20 aL. Number of released ions varied between 50 and 500 ions. Their behavior was assumed to be independent of each other. The average number of molecules inside the cleft at given time $Cu_{cleft}(t)$ was therefore proportional to the cumulative distribution of the single ion displacement over the 2 dimensional circle of radius R:

$$Cu_{cleft}(t) = Cu^T (1 - e^{-R^2/2Dt})$$

where Cu^T was the initial number of released Cu(II) ions.

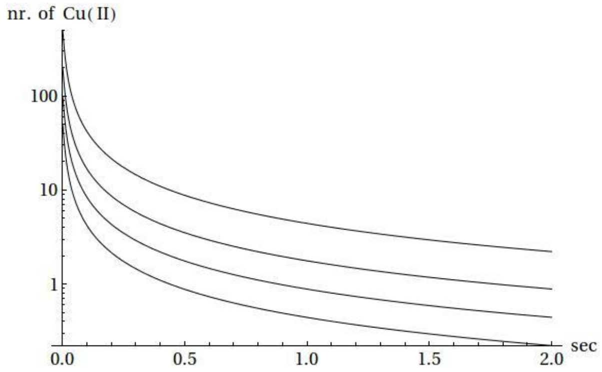

Figure A. The change in the number of Cu(II) ions inside the 2 aL, 5 aL, 10 aL and 20 aL (from the bottom to the top) volume upon the diffusion.

Bibliography

1. Nielsen TA, Digregorio DA, Silver RA, Street G, Kingdom U. Modulation of Glutamate Mobility Reveals the Mechanism Underlying Slow-Rising AMPAR EPSCs and the Diffusion Coefficient in the Synaptic Cleft. Neuron. 2004;42:757–71.

2. Waters J. The Concentration of Soluble Extracellular Amyloid-β Protein in Acute Brain Slices from CRND8 Mice. Smith MA, editor. PLoS One. 2010 Dec 29;5(12):e15709.

3. Vestergaard M, Hamada T, Saito M, Yajima Y, Kudou M, Tamiya E, et al. Detection of Alzheimer's amyloid beta aggregation by capturing molecular trails of individual assemblies. Biochem Biophys Res Commun. Elsevier Inc.; 2008 Dec;377(2):725–8.
