# Supplementary material for: Numerical Simulations Reveal Randomness of Cu(II) Induced Aβ Peptide Dimerization under Conditions Present in Glutamatergic Synapses
Source: PLoS One. 2017 Jan 26;12(1):e0170749. doi: 10.1371/journal.pone.0170749 (PMC5268396; doi:10.1371/journal.pone.0170749)
Supplement: S3 Text — (PDF) [file pone.0170749.s014.pdf]

S3 Text. One molecule noise

In special cases when only two states are possible - 0 or 1 molecule of given specie - is possible, some simplifications can be made. Assuming that (at one fixed time point) the probability that there is exactly one molecule in the system is ***p***, the expected value and the standard deviation can be easily calculated and consequently also Relative Standard Deviation, i.e. their ratio:

$$RSD = \frac{Standard\ deviation}{Expected\ value} = \frac{\sqrt{\boldsymbol{p}(1-\boldsymbol{p})}}{\boldsymbol{p}}$$

In cases when ***p*** is small (***p*** << 1) a RSD simplifies to:

$$RSD \approx \frac{\sqrt{\boldsymbol{p}}}{\boldsymbol{p}} = \frac{1}{\sqrt{\boldsymbol{p}}}$$

i.e. boosts to infinity when ***p*** tends to 0.

This estimation extends to situations, when the probability of occurrences of states different from 0 or 1 is negligible.
